# Heparin-induced thrombocytopenia post-cardiovascular interventional therapy: a case report

**DOI:** 10.1186/s12872-022-02796-2

**Published:** 2022-08-03

**Authors:** Lishui Shen, Xiaohua Liu, Lian Chen, Xiaofei Gao, Yizhou Xu

**Affiliations:** Department of Cardiology, Affiliated Hangzhou First People’s Hospital, Zhejiang University School of Medicine, No. 261 Huansha Road, Shangcheng District, Hangzhou, 310016 China

**Keywords:** Heparin-induced thrombocytopenia, Hemorrhage, Platelet transfusion, Cardiovascular interventional procedure

## Abstract

**Background:**

Heparin-induced thrombocytopenia (HIT) is an antibody-mediated adverse drug reaction characterized by thrombocytopenia and thromboembolism. Herein, we present a case of HIT with subcutaneous hemorrhage after cardiovascular interventional therapy.

**Case presentation:**

A 74-year-old man was admitted to the hospital for elective atrial fibrillation (AF) catheter ablation and left atrial appendage closure because of intermittent dizziness and palpitations. At presentation, the routine laboratory test results showed no abnormalities. He received subcutaneous enoxaparin for stroke prevention and unfractionated heparin for intraprocedural anticoagulation during coronary angiography and the AF procedure. On the second day after the AF procedure, the patient developed profound thrombocytopenia, moderate anemia, and mild subcutaneous hematoma. Blood tests and imaging examinations excluded acute hemolysis and other active bleeding. A 4Ts score of 5 and markedly positive platelet factor 4 IgG antibody established the diagnosis of HIT. Due to progressive subcutaneous hemorrhage in the thighs that could not be suppressed by pressure dressing, the patient received platelet transfusion and rivaroxaban for anticoagulation. The following days, the patient remained clinically stable from the hemorrhage, and his platelet count recovered. No thrombotic events occurred during hospitalization or follow-up.

**Conclusion:**

This case emphasizes the significance of suspecting HIT in patients with unexplained rapid thrombocytopenia after frequent heparin exposure. Decision-making regarding alternative anticoagulation and platelet transfusion in HIT with hemorrhage must be based on unique patient characteristics.

## Background

Heparin-induced thrombocytopenia (HIT) is a rare but important cause of morbidity and mortality in patients exposed to heparin [1]. As an immunologic condition, HIT is induced by IgG antibodies recognizing complexes of platelet factor 4 (PF4) and heparin and presents with thrombocytopenia and thrombosis [2]. Typically, HIT develops 5–14 days after heparin administration and presents with a mild-to-moderate reduction in platelet count [3]. Herein, we describe a case of HIT with significant thrombocytopenia and subcutaneous hemorrhage after cardiovascular interventional therapy.

## Case presentation

A 74-year-old man with a 10-year history of atrial fibrillation (AF) and a 5-year history of hypertension was brought to our hospital because of dizziness and palpitations. He had a 4-year history of intermittent dizziness, chest distress, and palpitations after exertion. Three years ago, he developed a transient ischemic attack and started anticoagulation therapy with warfarin; however, his international normalized ratio of prothrombin time often fluctuated. One week before admission, the patient developed spontaneous gingival bleeding, and warfarin was discontinued.

**Fig. 1:**
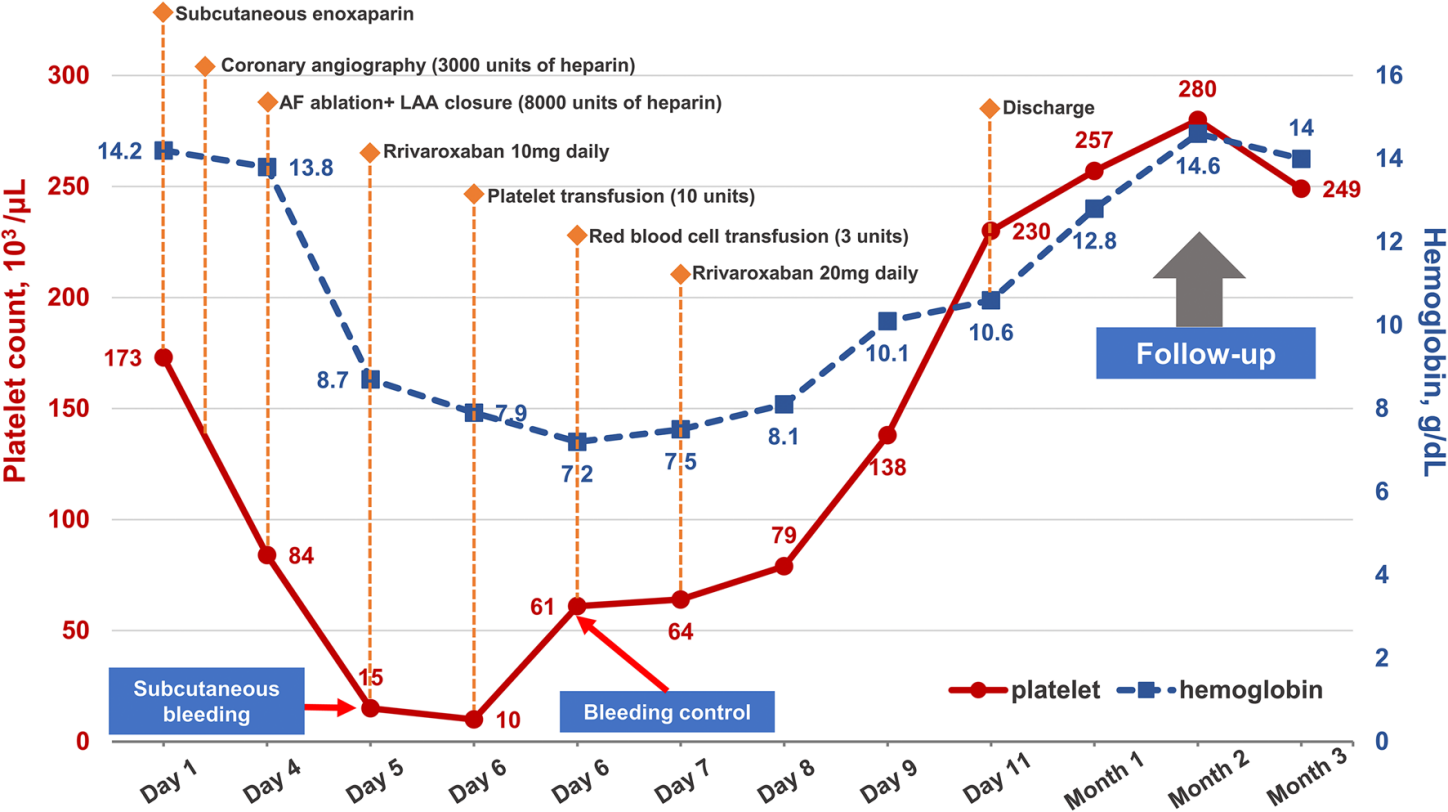
Platelet count, hemoglobin level, hemorrhagic events and treatment in a patient with HIT after cardiovascular interventional therapy. *AF* atrial fibrillation, *HIT* heparin-induced thrombocytopenia, *LAA* left atrial appendage

**Fig. 2:**
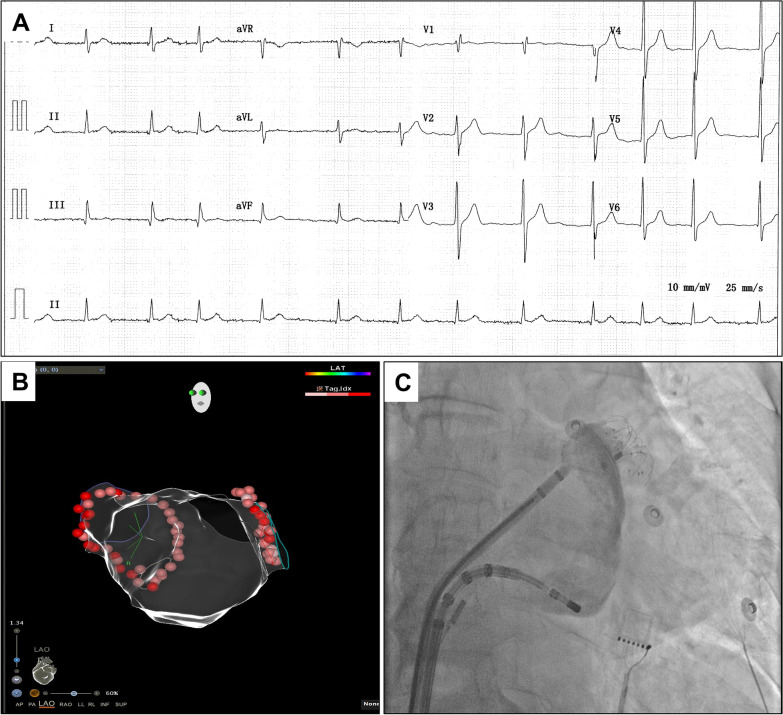
The elective atrial fibrillation procedure. Electrocardiography indicated the atrial fibrillation rhythm (**A**). The patient performed radiofrequency pulmonary vein isolation (**B**) and left atrial appendage closure (**C**)

At presentation, his blood pressure was 132/82 mmHg and heart rate was 80 beats per minute (bpm). His blood routine test results were within the normal limits (platelet count of 173 × 10^3^/µL [normal 125–350 × 10^3^/µL], red blood cell count of 4.3 × 10^6^/µL [normal 4.3–5.8 × 10^6^/µL], and hemoglobin level of 14.2 g/dL [normal 13.0–17.5 g/dL]) (Fig. 1). Electrocardiography showed persistent AF with an average heart rate of 72 bpm (Fig. 2A). Electrocardiographic monitoring revealed a markedly increased heart rate of nearly 140 beats/min after mild activity. The patient was administered a subcutaneous injection of low-molecular-weight heparin (enoxaparin) for stroke prevention at a fixed dose of 40 mg every 12 h. Due to the presence of stenosis in the right coronary artery (RCA) revealed by computed tomographic angiography, the patient further underwent coronary angiography, which demonstrated 50% stenosis in the RCA. During angiography, he received 3000 units of intravenous heparin for anticoagulation and reported no periprocedural complications.

Considering the high risk of thromboembolism (CHA2DS2-VASc score of 4) and hemorrhage (HAS-BLED score of 3), the patient was scheduled for AF ablation and left atrial appendage (LAA) closure. Before the AF procedure, transesophageal echocardiography (TEE) examination precluded an intracardiac thrombus. On day 4, he underwent successful radiofrequency pulmonary vein isolation under the guidance of an electroanatomic mapping system (CARTO-3, Biosense Webster) and intracardiac echocardiography (Biosense Webster), and an LAA occluder was placed (LAmbre, Lifetech Scientific) with no detectable LAA blood reflux after implantation (Fig. 2B, C). During the procedure, he received 8000 units of heparin with an activated clotting time of 300–350 s. Immediately returning to the ward, he experienced significant hypotension with systolic blood pressure in the 70 s. Echocardiography revealed normal cardiac function and excluded pericardial effusion. Blood tests showed a decreased platelet count of 84 × 10^3^/µL, and normal hemoglobin (13.8 g/dL), fibrinogen (2.15 g/L; normal 2.0–4.0 g/L), and d-dimer (570 µg/L; normal ≤ 500 µg/L) levels. After intravenous fluid resuscitation, the patient regained normal blood pressure and continued subcutaneous enoxaparin for anticoagulation therapy.

**Fig. 3:**
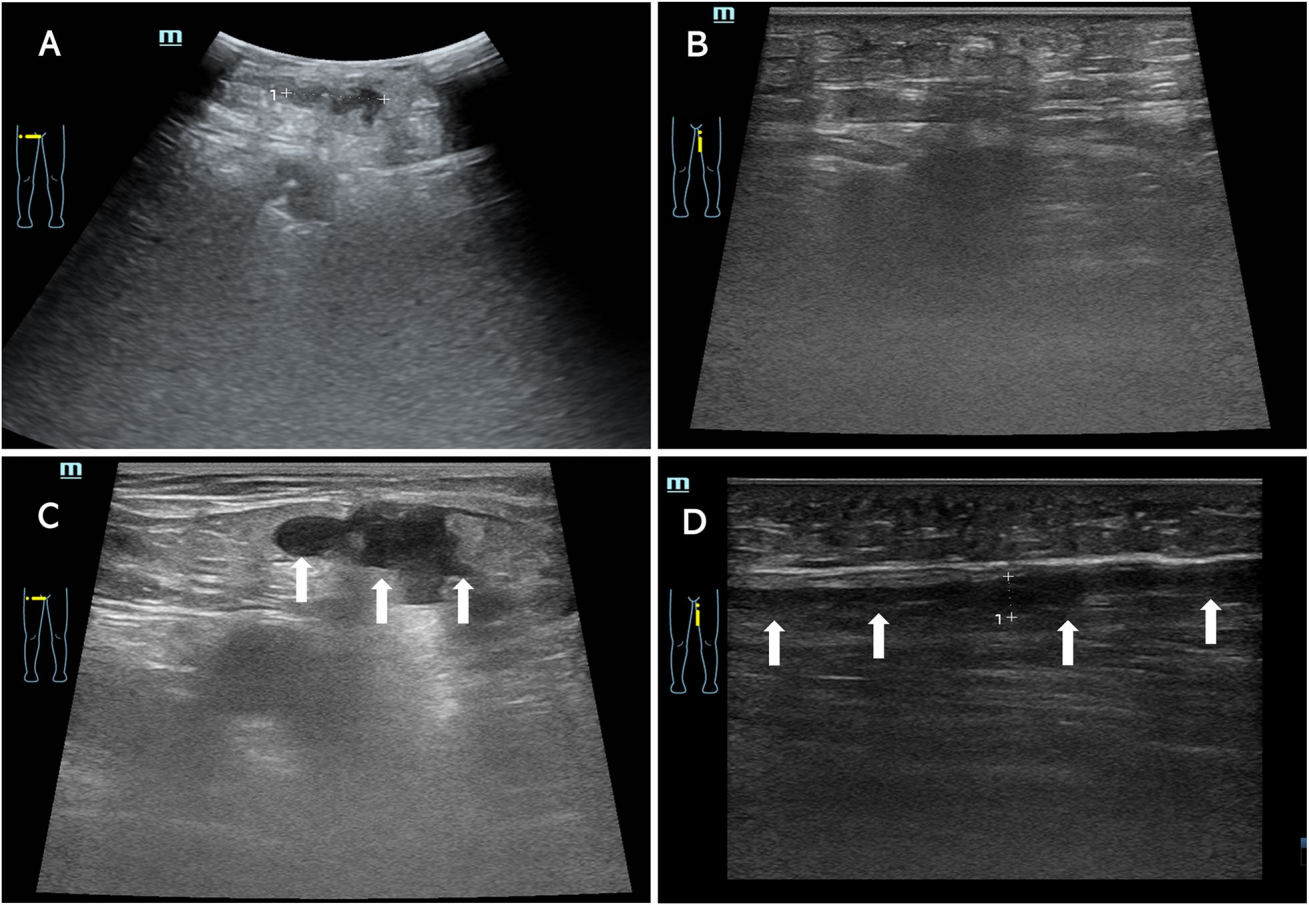
Lower extremity ultrasound images of the HIT patient. The initial ultrasound examination only showed a localized hematoma in the right thigh (**A**) with no significant hemorrhage sign in the left thigh (**B**). The second ultrasound examination indicated the enlarged hematoma in the right thigh (**C**) and new developed subcutaneous hemorrhage in the left thigh (**D**)

On day 5, the patient experienced progressive fatigue and dizziness. The patient denied melena, hematuria, or hematemesis. Physical examination revealed no dermal ecchymosis, hematoma, or vascular murmurs. Laboratory tests revealed a markedly decreased platelet count of 15 × 10^3^/µL, red blood cell 2.6 × 10^6^/µL and hemoglobin 8.7 g/dL, elevated white blood cell of 11.0 × 10^3^/µL (normal 3.5 × 10^3^/µL), normal red blood cell morphology, and negative red blood cell irregular antibody (Fig. 1). Further tests excluded the possibility of acute hemolysis. Computed tomography and ultrasonic examination excluded active bleeding from the brain, chest, abdomen, and pelvis. Lower extremity ultrasonography revealed a localized subcutaneous hematoma in the right thigh with no signs of pseudoaneurysm, arteriovenous fistula, or arterial/venous thrombosis (Fig. 3A, B). Hematology was consulted, and HIT was suspected with a 4Ts score of 5 (1 point for platelet nadir 10 × 10^3^/µL; 2 points for platelet fall within 1 day and prior heparin exposure within 30 days; 2 points for no apparent other causes of thrombocytopenia). Further, heparin PF4 antibody test indicated a positive result with an OD of 2.150, which established the diagnosis of HIT.

Given the high risk of thromboembolism due to HIT and post-AF procedure status, the patient discontinued enoxaparin and cautiously started rivaroxaban (initial dose of 10 mg daily) for anticoagulation. He received a pressure dressing to suppress the subcutaneous hematoma. However, the hematoma enlarged, and his left thigh developed a patchy subcutaneous hemorrhage (Fig. 3C, D). The platelet count further decreased to 10 × 10^3^/µL. Considering the progressive blood loss and the difficulty of hemostasis, 3 units of suspended red blood cells and 10 units of platelets were cautiously administered. Fortunately, his subcutaneous hemorrhage was well controlled, and no thrombotic events occurred after platelet transfusion. The platelet count recovered to 138 × 10^3^/µL and hemoglobin to 10.1 g/dL three days after therapy. The rivaroxaban dose was increased to 20 mg daily before discharge. Based on the recommendations of the Chinese expert consensus statement on LAA closure [4], the patient was treated with rivaroxaban 20 mg daily for the initial 3 months. At the 3-month follow-up, the patient remained in sinus rhythm and reported no occurrence of thrombocytopenia, hemorrhage, or thromboembolism. A repeat TEE test showed no device-related thrombus or peri-device leakage. He was administered aspirin 100 mg daily and clopidogrel 75 mg daily for another 3 months, followed by a single aspirin dose of 100 mg daily for long-term therapy.

## Discussion and conclusions

Heparin has been used as a first-line anticoagulant in interventional cardiovascular procedures. However, prior heparin exposure can sometimes lead to severe complications such as HIT, which increases the risk of both thrombosis and hemorrhage. Herein, we reported a case of HIT that presented with thrombocytopenia and significant subcutaneous hemorrhage after AF ablation and LAA closure.

HIT is an immunological condition caused by pathological IgG antibodies against PF4)/heparin complexes. Immune complexes can bind to and activate FcγRIIA receptors on platelets, endothelial cells, and monocytes, resulting in a hypercoagulable state and thrombosis. Typical HIT presents an approximately 50% decrease in platelet count 5–14 days after initiation of heparin and can lead to thromboembolic complications in 35–75% of patients [5, 6]. The 4Ts score is a widely used scoring system for estimating the probability of HIT [7]. In this case, the patient had a definite history of heparin use, and a 4Ts score of 5 indicated an intermediate probability of HIT. Considering his positive heparin-PF4 antibody test result, a HIT diagnosis was established. Notably, the initial reduction in the platelet count in this case did not attract our attention. Continuation of heparin treatment resulted in profound thrombocytopenia and subcutaneous hemorrhage. Spontaneous bleeding is uncommon in HIT, while severe thrombocytopenia can increase the risk of hemorrhage [8]. Our patient underwent invasive procedures, including AF ablation and LAA closure, during which an 11-F sheath and a 6-F sheath were inserted into the left femoral vein, and two 8.5-F Swartz sheaths and a 12-F sheath into the right femoral vein. These vascular puncture procedures contributed to the bleeding event, and conventional pressure dressing showed limited value for bleeding control owing to the extremely low platelet count. Previous studies reported that administering a platelet transfusion to a patient with HIT seemed to “add fuel to the fire,” while a recent study demonstrated no thrombotic complications in all 37 HIT patients following platelet transfusion [9]. Similarly, our case demonstrates the safety and efficacy of platelet supplements for refractory hemostasis in HIT. However, owing to the potential thrombotic risk in HIT, our patient still required anticoagulation therapy. The recommended dose of rivaroxaban for isolated HIT was initially 15 mg twice per day until platelet recovery, then 20 mg daily [10]. Currently, there is no recommended dose for patients with HIT and active bleeding. For safety reasons, the patient was initially administered low-dose rivaroxaban (10 mg daily). In clinical practice, the combined therapy of cautious anticoagulation and platelet transfusion may serve as a useful treatment for patients with HIT and active bleeding; however, its safety remains unclear.

Another significance of this case is that it emphasizes the importance of the early recognition of HIT. Although the clinical prevalence of HIT is less than 5% in patients receiving therapeutic doses of heparin, its high mortality (nearly 10%) and disability, typically a result of thromboembolic complications, significantly affect the safety of cardiac surgery and interventional therapy [11]. In high-volume heart centers, short hospital stays and lack of timely blood reexamination may increase the probability of a missed diagnosis of HIT, especially in the population undergoing multiple interventional therapies. Moreover, the late occurrence of platelet count reduction in classical (at least 5 days after heparin administration) and delayed-onset HIT (from 5 days to 3 weeks after heparin is discontinued) also promotes the ignorance of this complication [12]. Our patient underwent coronary angiography, AF ablation, and LAA closure in a single hospitalization period, during which he frequently received unfractionated and low-molecular-weight heparin for anticoagulation. Fortunately, his delayed discharge due to a postoperative hypotension event allowed us to identify potential HIT and hemorrhage in a timely manner.

In summary, clinicians should consider HIT in patients with unexplained rapid thrombocytopenia, especially within the expected time course following heparin exposure. Decision-making regarding alternative anticoagulation and platelet transfusion must be based on unique patient characteristics.

## Data Availability

All data supporting the conclusions are presented in the manuscript.

## Abbreviations

AF: Atrial fibrillation
BPM: Beat per minute
HIT: Heparin-induced thrombocytopenia
LAA: Left atrial appendage
